# Women's knowledge and beliefs regarding breast cancer

**DOI:** 10.1038/sj.bjc.6600260

**Published:** 2002-05-06

**Authors:** E A Grunfeld, A J Ramirez, M S Hunter, M A Richards

**Affiliations:** Psychology Unit, Guy's, King's and St Thomas' Medical School, Guy's Campus, London SE1 9RT, UK; Section of Liaison Psychiatry and ICRF Psychosocial Oncology Group, Guy's, King's and St Thomas' Medical School, St Thomas' Hospital, London SE1 7EH, UK

**Keywords:** breast cancer, symptoms, risk factors, age

## Abstract

Approximately 20–30% of women delay for 12 weeks or more from self-discovery of a breast symptom to presentation to a health care provider, and such delay intervals are associated with poorer survival. Understanding the factors that influence patient delay is important for the development of an effective, targeted health intervention programme to shorten patient delay. The aim of the study was to elicit knowledge and beliefs about breast cancer among a sample of the general female population, and examine age and socio-economic variations in responses. Participants were randomly selected through the Postal Address File, and data were collected through the Office of National Statistics. Geographically distributed throughout the UK, 996 women participated in a short structured interview to elicit their knowledge of breast cancer risk, breast cancer symptoms, and their perceptions of the management and outcomes associated with breast cancer. Women had limited knowledge of their relative risk of developing breast cancer, of associated risk factors and of the diversity of potential breast cancer-related symptoms. Older women were particularly poor at identifying symptoms of breast cancer, risk factors associated with breast cancer and their personal risk of developing the disease. Poorer knowledge of symptoms and risks among older women may help to explain the strong association between older age and delay in help-seeking. If these findings are confirmed they suggest that any intervention programme should target older women in particular, given that advancing age is a risk factor for both developing breast cancer and for subsequent delayed presentation.

*British Journal of Cancer* (2002) **86**, 1373–1378. DOI: 10.1038/sj/bjc/6600260 www.bjcancer.com

© 2002 Cancer Research UK

## 

Delayed presentation of symptomatic breast cancer of three months or more is associated with lower survival rates (Richards *et al*, 1999a). While some of this delay is health-provider related, an estimated 20–30% of women wait at least three months before seeking medical help for breast symptoms (Richards *et al*, 1999b). A recent fall in deaths from breast cancer has been reported due to improved survival from a combination of earlier diagnosis, breast screening and improvement in treatment methods (Richards *et al*, 2000; Peto *et al*, 2000). The relative contribution of these factors remains to be clarified. In the meantime, breast cancer continues to represent a major public health problem, and further gains in survival might be achieved by encouraging women to seek help more promptly. Understanding the factors that influence patient delay is a prerequisite for the development of strategies to shorten delays. Strong evidence suggests that older women are more likely to delay their presentation with breast cancer, although the strength of evidence for other risk factors for delay is inadequate to inform any intervention (Ramirez *et al*, 1999). Such factors are likely to relate to women's knowledge and beliefs about breast cancer and its management.

The NHS Breast Screening Programme aims to invite all women aged 50–64 for mammography screening, and the uptake of invitations for screening is high (Department of Health, 2000a). The Department of Health now plans to extend routine breast screening to women up to the age of 70, and the procedure will be available on request to women over 70 (Department of Health, 2000b). It has been estimated that an effective screening programme may reduce mortality in the screening age group by up to 25% (Blamey *et al*, 2000). However women still need to be ‘breast aware’ and to accurately identify breast symptoms in order to receive treatment as quickly as possible (as symptoms may develop between screening appointments). Furthermore, approximately 44% of breast cancer cases occur in women within the screening age range; 21% of cases occur in women under the age of 50 and a further 35% of cases occur in women aged over 70 years (Office for National Statistics, 2000). Therefore, women outside of the routine screening age group will still need to be informed of the risks and symptoms of breast cancer.

Women's knowledge of breast cancer risk factors and of survival have been studied in Scottish, American and Australian populations (Roberts *et al*, 1984; Breslow *et al*, 1997; Paul *et al*, 1999). No studies, however, have employed a UK national perspective to examine women's knowledge of the risks associated with breast cancer and their perceptions of management and outcomes associated with the disease. Furthermore, there are currently no large sample studies examining women's knowledge of the range of breast symptoms. Representative and up-to-date data are essential to target educational resources to the women most at need, across a broad age and socio-economic status (SES) range. The aims of the present study were to (1) develop this knowledge base through the examination of women's interpretations of potential symptoms of breast cancer, their beliefs about of the risks and consequences of breast cancer and, (2) examine these variables in relation to age and SES.

## MATERIALS AND METHODS

Sampling and data collection were conducted by the Office of National Statistics (ONS) as a module of their Omnibus survey. Data collection took place during January and February 2000. Three thousand addresses were selected from the Postal Address File (PAF) and a letter sent to each household outlining the survey. Trained interviewers visited each address and at least three calls were made, at different times of the day and week. The interview schedule was specifically designed for the study, and pilot testing was conducted to ensure the survey was comprehensible to the target group. The questions examined four issues:

1. *Knowledge of a woman's lifetime risk of developing breast cancer:* Participants were first asked a knowledge question and were required to choose a woman's approximate overall lifetime risk (approximately 1 in 3, 10, 100, 1000, 10 000) of developing breast cancer. They were then asked to provide a general estimation of their personal risk in comparison to the general population and were required to state whether they perceived themselves to be more, less, or as likely to develop breast cancer than the rest of the female population as a whole.
2. *Knowledge of the risk factors associated with breast cancer:* Participants were shown a list of 10 established or probable risk factors and six non-risk factors (Royak-Schaler *et al*, 1996). The items in the list were presented in a random order of established and non-risk factors. They were asked to choose those that they believed would increase a woman's chance of developing breast cancer. The percentage of respondents reporting each factor as a risk factor is reported. A score was produced of the number of correct risk factors identified by each respondent (range 0–10).
3. *Knowledge of breast cancer symptoms:* Participants were shown a card containing 12 breast changes (eight of which were potential breast cancer symptoms) and asked to indicate which they thought could be potential symptoms of breast cancer. (The order of the breast changes was randomly assigned.) The percentage of respondents reporting each symptom as a potential symptom of breast cancer is reported. A score was produced of the number of correct potential symptoms identified by each respondent.
4. *Perceptions of management and outcomes in breast cancer:* Participants were asked to identify methods of treatment used for breast cancer and the responses were categorised according to themes. Participants rated attitudinal statements about the efficacy and consequences of breast cancer treatment on a four-point scale (strongly agree to strongly disagree). The lower the score the more the participant agreed with the statement.

### Participants

From the original 3000 randomly chosen addresses there were 1830 respondents (a response rate of 67%). Nine hundred and ninety-six of these respondents were female (mean age 47 years, range 16–96) and completed the breast cancer module of the survey. The demographic groupings of the sample can be found in Table 1

**Table 1:**
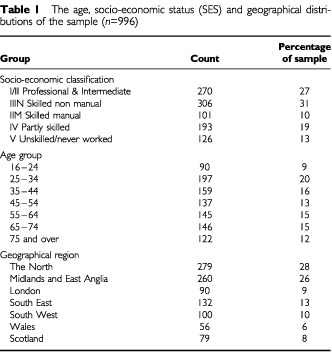
The age, socio-economic status (SES) and geographical distributions of the sample (*n*=996)

. Information regarding non-responders is not available due to the nature of the data collection, however, the age, SES and geographical distributions of the data are representative of the UK population.

### Statistical analysis

The data were analysed according to the age and SES groups shown in Table 1. Response frequencies are summarised as percentages. Chi-square tests were used to analyse the categorical and attitudinal responses according to the groups. Multivariate analysis of variance was used to examine group differences in response to the scale data (total risk score and total symptom score).

## RESULTS

### Risk estimation for developing breast cancer

Respondents were overly optimistic regarding a woman's risk of developing breast cancer, with 31% reporting that a woman had a 1 in 1000 chance of developing breast cancer, 35% reporting a one in 100 risk and 23% correctly indicating a 1 in 10 risk. The majority of the sample (76%) reported that they were just as likely to develop breast cancer as the rest of the female population in the UK, while 7% reported a belief that they were at increased risk, and 17% reported that they were less likely to develop breast cancer. However, there was a relationship between age and the perception of risk (χ^2^=54.27, df=6, *P*<0.001) with 35% of over 65-year-olds and 30% of over 75-years-olds reporting reduced personal risk. There was also a relationship between SES and perception of risk (χ^2^=46.11, df=4, *P*<0.001), with 32% of professional and intermediate (non-manual, non-managerial occupation) women reporting reduced risk compared to 10–15% of partly skilled and unskilled women. The explanations for reduced risk (in response to an open-ended question) provided by the women included absent family history (47%), lifestyle factors, such as following a healthy diet and exercise plan (12%) and not smoking (5%). Approximately 25% of the women reporting a belief that they were at reduced risk believed that they were too old to develop breast cancer; the mean age of this sub-sample was 76 years (range 63–91). These participants provided explanations such as: ‘I think if I was going to develop it I would have by now’, ‘At my age I think the danger has passed’, ‘[Because] they stop mammograms at 65’ and ‘I think older people are less likely to have breast cancer problems’.

### Risk factors for breast cancer

A family history of breast cancer and a personal history of breast cancer were the most frequently cited risk factors, whereas less than one third recognised the role of advancing age (Table 2

**Table 2:**
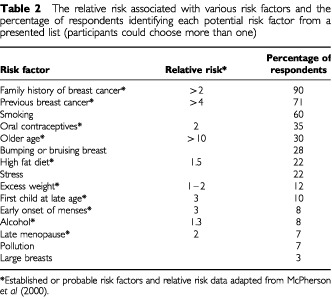
The relative risk associated with various risk factors and the percentage of respondents identifying each potential risk factor from a presented list (participants could choose more than one)

). Table 2 shows the number of correct risk factors identified according to age group. Women over 75 correctly identified fewer risk factors than women aged under 65 (F(6, 903)=13.22, *P*<0.001). Professional women and women classified as intermediate (SES groups I and II) had a greater knowledge of risk factors than women who were partly skilled, skilled manual workers, or who had never worked (F(4, 1070)=17.50, *P*<0.001). Women aged 35–59 were perceived, by the sample, to be most at risk of developing breast cancer, and those aged 75–90 years to be at least risk (Figure 1

**Figure 1:**
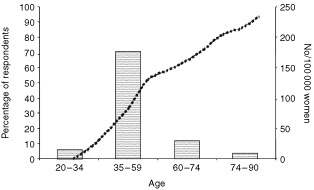
The percentage of respondents perceiving each of the age categories shown to be most at risk of developing breast cancer (bars). The dotted line (corresponding to the right hand axis) represents an approximation of the incidence of breast cancer according to each age category (adapted from McPherson *et al*, 2000).

).

### Symptoms of breast cancer

A painless breast lump, lump under the armpit, and nipple discharge were the most frequently identified symptoms of breast cancer (Table 3

**Table 3:**
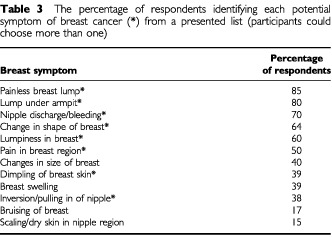
The percentage of respondents identifying each potential symptom of breast cancer (*) from a presented list (participants could choose more than one)

). Less than half of the sample identified dimpling of the breast skin and nipple inversion as signs of breast cancer. The oldest age cohort identified fewer types of symptoms of breast cancer than women aged between 25 and 74 (F(6, 919)=10.64 *P*<0.001; Table 4

**Table 4:**
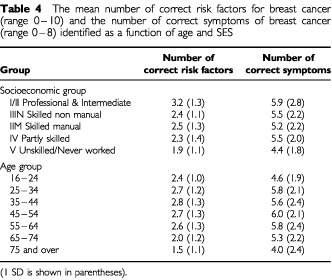
The mean number of correct risk factors for breast cancer (range 0–10) and the number of correct symptoms of breast cancer (range 0–8) identified as a function of age and SES

). Women who were unskilled or had never worked identified significantly fewer symptoms than the other socio-economic groups (F(4, 1069)= 10.43, *P*<0.001; Table 4).

### Management of breast cancer

The most frequently cited treatment method for breast cancer was surgery (87%), followed by chemotherapy (66%) and radiotherapy (49%). Only 5% of the sample spontaneously mentioned hormone therapy as a treatment method. Tables 5

**Table 5:**
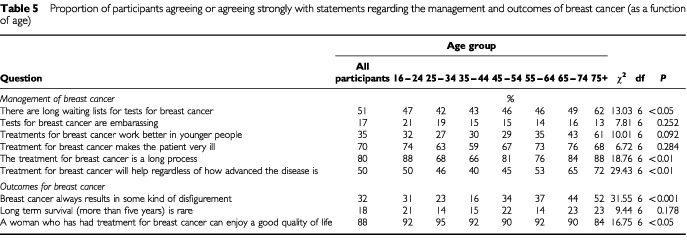
Proportion of participants agreeing or agreeing strongly with statements regarding the management and outcomes of breast cancer (as a function of age)

and 6

**Table 6:**
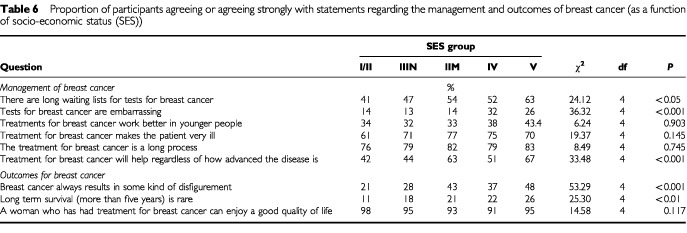
Proportion of participants agreeing or agreeing strongly with statements regarding the management and outcomes of breast cancer (as a function of socio-economic status (SES))

show the responses to the attitudinal questions of the management of breast cancer as a function of age and SES. Eighty per cent of the women believed treatment for breast cancer to be a long process that would make a patient very ill. Approximately 50% of participants thought that there were long waiting lists for tests for breast cancer, with those over 75 years old more likely to report this belief than participants under 45 (χ^2^=13.03, df=6, *P*<0.05).

### Outcomes of breast cancer

Tables 5 and 6 show the responses to the attitudinal statements regarding outcomes associated with breast cancer, as a function of age and SES. Approximately, 80% of participants believed long-term survival (greater than five years) to be common, and 70% did not believe the disease necessarily resulted in disfigurement. However, women aged over 75 were more likely to believe that disfigurement was a frequent outcome of breast cancer (χ^2^=31.55, df=6, *P*<0.001), as were manual and unskilled workers (χ^2^=53.29, df=4, *P*<0.001). Professional and intermediate women (SES groups I and II) were more likely to believe that five-year survival following breast cancer was achievable (χ^2^=25.30, df=4, *P*<0.01).

## DISCUSSION

The results of the survey demonstrated that although British women have good understanding of some aspects of breast cancer there is poor awareness of other important issues, including knowledge of non-lump breast symptoms and lifetime risk of developing the disease. The survey also highlighted important age and SES variations in knowledge of risk and of the range of potential symptoms of breast cancer. These variations may help to explain some of the differences in help-seeking behaviour observed among women with breast cancer symptoms in the UK.

### Risk perception

Respondents were overly optimistic regarding a woman's risk of developing breast cancer with less than one quarter correctly indicating a 1 in 10 risk. Although health education campaigns have included information about the lifetime risk of breast cancer, it is apparent that the majority of British women have either not accessed this information or have not interpreted it correctly. However, the format of these educational campaigns may also account for the poor awareness observed among these women. It is known that leaflets produce only limited and short-lived changes in knowledge (Gatherer *et al*, 1979). Furthermore, many health professionals believe leaflets are often not read by the target audience (Murphy and Smith, 1993). Therefore, any future campaign will need to make explicit the significant risk that breast cancer poses for women and combine the more traditional leaflet approach to health education with other educational mediums (i.e. television and radio broadcasts) and individually tailored advice from health professionals.

The findings of this study suggest that the importance of advancing age as a risk factor for breast cancer is poorly understood, not only by older women in this country, but by the general female UK population as a whole. This is in line with findings from USA and Australian populations (Breslow *et al*, 1997; Dolan *et al*, 1997; Paul *et al*, 1999). In the current sample, women aged 35–59 years were perceived to be most at risk of developing breast cancer. Breast cancer is the single commonest cause of death among women aged 40–50, however, in absolute terms advancing age is the greatest risk factor for developing breast cancer (McPherson *et al*, 2000); approximately one-third of all breast cancers occur in women aged over 70 (Office for National Statistics, 2000).

In our sample, a significant proportion of women aged over 65 perceived themselves to be at less personal risk than the general population, and in a significant proportion of these the explanation for reduced risk related to their advanced age. In the UK, women aged 50–64 are routinely invited for breast cancer screening (Department of Health, 2000a) and this may contribute to the increased risk attributed to this age group. Although not directly examined in the present study, the results suggest that by stopping screening at 64 a message may inadvertently be sent to women that they are no longer at risk, in fact one respondent explicitly stated this belief. This is a concern that has been expressed previously (Age Concern, 1996). The Department of Health plans to extend routine screening to all women up to the age of 70 by 2004 (Department of Health, 2000b), as there is evidence that screening is acceptable in the age group 65–70 and is likely to save lives. Screening will also be available on request to women over 70. The results suggest that consideration should be given to the best way of communicating the need for continuing breast awareness among women over 70.

In addition to poor awareness of advancing age as a risk factor, older women demonstrated poorer knowledge of risk factors in general. This lower level of knowledge was also apparent among women in SES groups III and IV. Surveys in the USA and Australia have demonstrated that older women, particularly those classified as lower SES, have poorer knowledge of key risk factors for various cancers (Paul *et al*, 1999; Breslow *et al*, 1997). Additionally, professional women in the present study perceived themselves to be at reduced risk in comparison to the rest of the female population. However, there is evidence to suggest that this group may be at increased risk of breast cancer due to a combination of lifestyle factors (Wagener and Schatzkin, 1994). The results of the present study suggest therefore, that information regarding risk factors and personal risk should also be targeted across SES groups.

One risk factor that the majority of women recalled was a family history of the disease, and this finding is in agreement with previous research (Paul *et al*, 1999). Although women with a strong family history of breast cancer have a higher risk, a larger percentage of cases occur in women without a positive family history (McPherson *et al*, 2000). This emphasis on family history as a risk factor for breast cancer could potentially lead to a state of complacency among women for whom there is no known family history. It was outside the scope of the present study to examine this hypothesis, however, previous research has demonstrated that women with a family history may overestimate their risk of developing disease (Evans *et al*, 1993; Neise *et al*, 2001). Furthermore, increased personal risk perception may have a negative effect on participation in breast screening (Neise *et al*, 2001), suggesting that consideration should be given to the way that information is presented even to women at increased risk.

### Knowledge of breast cancer symptoms

The majority of the women surveyed recognised a painless breast lump as a symptom of breast cancer. In line with previous findings (Facione, 1993), less than half the sample recognised dimpling of the breast skin, nipple retraction or nipple eczema as symptoms of breast cancer. All of these conditions, however, are considered to warrant hospital referral in a significant proportion of women (Dixon and Mansel, 1994; Fentiman and Hamed, 2001). These findings confirm previous qualitative research with women with breast cancer, which has demonstrated that non-lump breast symptoms are less likely to be attributed to breast cancer (Burgess *et al*, 1998, 2001). The results are important, as there is moderate evidence to suggest that one of the major determinants of delay behaviour among patients is the discovery of a breast symptom other than a lump (Ramirez *et al*, 1999). In line with previous work (Facione and Dodd, 1995) our results demonstrate that although a breast lump is equated with a potential cancer, other potentially serious symptoms may be misinterpreted. Detailed analysis of women's interpretation of individual symptoms is necessary in order to determine women's perceptions and attributions of breast symptoms. The summary message is that women would benefit from clear information about the variety of symptoms that may be indicative of a potential cancer. However, any intervention to improve knowledge of symptoms should also aim to limit anxiety and to ensure that medical facilities are not overloaded by help-seeking for benign symptoms, particularly by low risk women.

Knowledge of symptoms was poorer among older women and women who had never been employed. Older women were less likely to perceive nipple eczema, changes in the shape or size of the breast, and nipple retraction as symptoms of breast cancer. It is possible that older women attribute such symptoms to the ageing process, as has been reported previously for other symptoms (Leventhal and Prohaska, 1986). Furthermore, it has been argued that older adults, who may have a number of symptoms of other illnesses, should not be expected to seek help for symptoms that are not causing them pain or that have little effect on their functioning (Ford and Taylor, 1985). The misattribution and limited handicap associated with early breast cancer symptoms may contribute to the delay observed among this age cohort. Therefore older women, in particular, may require further information regarding the potential seriousness of breast changes and recommendation for action if they identify such symptoms.

### Perceptions of treatment and consequences

The majority of participants reported that five-year survival was a common event, which reflects the improvements in five-year survival that have recently been reported (Welch *et al*, 2000). The majority of respondents, however, reported negative perceptions regarding the length of treatment and the accompanying side effects. It is probable that their perceptions related to surgical and chemotherapeutic treatment modalities, as these were the methods most frequently mentioned. This emphasis on surgery has been reported previously (Roberts *et al*, 1984). Hormone therapy was cited by only 5% of respondents, yet this is one of the most common post-operative treatments prescribed for older women (Bellet *et al*, 1995). Older women were more likely to perceive that treatments would work better in younger patients. However, there is no evidence of this and it is recommended that older women be treated with a similar protocol to younger patients (Dixon *et al*, 1994). Older patients may be excluded from treatment on the grounds of co-morbidity or functional disability, furthermore, there may also be problems with treatment due to difficulties accessing transport and poor compliance (Bellet *et al*, 1995). It is these factors that may influence perceptions of the efficacy of treatment among older women. The older group were also more likely to report that breast cancer would result in disfigurement, which may reflect this cohort's experience of peers who may have been treated at a time when methods were less effective and associated with poor aesthetic outcomes. Further research would be required to confirm the reasons why older women may hold more negative perceptions of the outcomes associated with breast cancer.

Women generally reported positive beliefs about breast cancer outcomes, however there were aspects of symptomatology and risk that were poorly understood. This poor level of knowledge could potentially contribute to delay in seeking medical help. This is especially true for older women, who have a poor awareness both of the risk factors and of the range of symptoms associated with breast cancer. This poor level of knowledge is of particular concern, given the increased risk of developing breast cancer with advancing age, and may partly explain the increased delay behaviour observed among older women. However, further research is necessary to confirm this. The current study forms part of a larger programme of research examining delay behaviour for breast cancer. This includes work to examine the relationship between the intention to seek help for breast symptoms and knowledge and beliefs about breast cancer in the general female population. A future study is being planned drawing upon these findings with a clinical sample of women who have recently sought medical help for breast cancer symptoms, with the aim of developing a health promotion intervention.
